# Bringing Up Students in the Healthy Lifestyle Multiplier Students Program, São Paulo, Brazil

**Published:** 2008-06-15

**Authors:** Moacyr Roberto Cuce Nobre, Rachel Lima Zanetta, Inês Lancarotte

**Affiliations:** R Cd Cagliori; Cognos – Health Education Ltda, São Paulo, Brazil; Heart Institute (InCor), University of São Paulo Medical School, Brazil

## Abstract

Health education practices are central to preventing heart disease. The great challenge remains how to promote the adoption of healthy behaviors that can reduce risk factors for heart disease. In São Paulo, Brazil, the Healthy Lifestyle Multiplier Students program is based on studies that train older students ("multipliers") as peer educators for younger students. These students influence each others' cultural development and psychosocial growth, encourage one another to diversify their social relationships, and transform teaching-learning experiences. This intervention focused on physical activity, healthy diet, and the health effects of cigarette and alcohol consumption.

## Introduction

Lifestyle-associated health risks still greatly challenge health educators in Brazil. Although health education is a curricular requirement, information alone is not enough to modify habits, and Brazilian schools have lacked a creative approach to behavioral change.

Our approach takes into account students' knowledge, health behaviors, and attitudes; in other words, we consider what the students know about health, what they should know about cardiovascular disease (CVD) prevention, what they should do to promote health, and how they should incorporate this understanding into their lives. Before the pedagogical intervention, we used questionnaires and written reports to identify the students' ideas and values related to the topics to evaluate their potential for learning and teaching. During training, the students developed teaching skills (based on the same content used for training teachers) for the interaction with their peers. We describe the experience of 80 students who worked with more than 1060 classmates from 4 public elementary schools in São Paulo, Brazil. We then discuss the methods we used to develop health education materials for schools so that other health professionals and interested laypeople can develop similar peer education programs (1).

## Improving Teachers' Skills

Our approach to teacher training assumes that people do not learn by a simple process of perceiving and incorporating information nor by merely acquiring facts. Learning should combine perception, affect (emotions), motor actions, and social relationships. We based the design of the course on the work of Paulo Freire (2). The pedagogical practice, focused on understanding the psychological, cognitive, affective, cultural, and social reality of adolescents, is based on the work of Lev Vygotsky (3).

The multiplier students' peer activities are based on these principles and are conducted through horizontal dialogue, using appropriate language, approaches, and forms of communication. The concept of dialogue in education is central to Freire' s work. The term *horizontal dialogue *indicates Freire's rejection of hierarchic models of communication (vertical dialogue) in favor of a participatory process between equals. The communication process must respect the knowledge and culture of each social group involved. True dialogue, he suggests, is essential to social change and the transformation of individuals into agents of change.

In this program, multiplier students are the agents of change. Sharing information about health benefits and risks associated with lifestyle is effective because of the influence that adolescents exert on their peers' attitudes and behaviors. The same influence or pressure of a group of friends that leads to the adoption of risk behaviors, such as smoking, can be directed to supporting resistance to peer pressure.

We trained the teachers with the collaboration of graduate and postgraduate students from the cardiology department of São Paulo University Medical School. The training consisted of 4-hour modules every 2 months for 2 years, using expositive classes, lectures (4), focused discussion, and group dynamics. The content was based on scientific knowledge about 4 health promotion themes: physical activity, healthy diet, smoking, and alcohol consumption. Teachers received specific training to recruit, select, and support the students, who later worked with younger students.

The major problem was the teachers' resistance to the way adolescents worked with their peers, using informal language without medical terminology. This issue persisted throughout the first year of the project, partly because the teachers were accustomed to the traditional, positivistic pedagogical method. As the project went on and results became evident, this obstacle was overcome by reflexive dialogue.

We gave the teachers information about health and program strategies to standardize the language and to use as a content reference for the different participating schools (Appendix). The booklets are available, in Portuguese, at http://www.cognos.med.br/pesec.

## Healthy Lifestyle Multiplier Students

The theoretical educational model used to develop the healthy lifestyle multiplier system is based on Paulo Freire's dialogical constructivist model (2). This model assumes that knowledge is social and that learning occurs during social interactions leading to knowledge transfer. Learners are active risk takers who accept challenges and understand how and why to learn. The turning point of this constructivist perspective is when what Freire described as the "Banking Model of Education," in which the "educator's role is to regulate the way the world enters into the students," is no longer useful (2).

The Healthy Lifestyle Multiplier Students program is based on studies that bring together children close in age and from different school grades to influence their cultural development and psychosocial growth and to encourage both sets of students to diversify their social relationships and change experiences. As Viner and Macfarlane explain, "Improving social abilities is effective in enabling young people to make independent choices about individual health-related behaviors. It may also improve young people's self-esteem" (5). The teachers provided guided supervision while the multiplier students worked out the material and methods that were used to train the adolescents to become facilitators or leaders (5). From a pedagogical point of view, the work among students from different grades represents a rich environment full of possibilities for interaction and original solutions for teaching/learning problems as well as an opportunity for exercising tolerance and accepting differences (6).

The transmission of knowledge is based on entertaining and educational techniques that consider concepts, myths, and truths about health. What is a risk factor? What are the risks associated with lifestyle? What is CVD? What is the role of smoking and alcohol in the development of CVD? What is the importance of a healthy diet? What are the benefits of physical activity? How do calorie intake and energy consumption determine body mass?

We trained participating students as peer educators in collaboration with previously trained teachers from the selected schools. Students from an upper grade were trained to work on healthy lifestyle behaviors with students from the previous grade at a ratio of 3 multiplier students to each 30-student class. Teachers selected the multiplier students on the basis of their personal skills, leadership, and interest. At the end of 3 years, the multiplier students completed eighth grade (the last year of elementary school), and the younger students finished seventh grade.

The interaction among multiplier students and their peers occurred at 1-hour weekly meetings during the intervention period. The study sample was representative of the lower middle-class population of urban São Paulo, having an ethnic background that is 57% white, 26% mulatto, 11% black, and 6% other. The population was 58% male.

The learning content was arranged through concepts, skills, and attitudes — in other words, according to what the students know, should know, should know how to do, and should be (7). Before the pedagogical intervention, teachers observed students and discovered the importance the students placed on each theme (physical activity, healthy diet, smoking, and alcohol consumption). Next, we evaluated the teaching potential of the students by observing their performance and leadership skills. During their training, the students developed the didactic tools (scripts, board games, and lyrics) they would use in the multiplier activities. The tools are based on the content used for the teachers' training, but students are allowed to use their own informal language.

The goal of supervising the multiplier students was to enable them to support the dialogue with their peers on health and prevention of CVD, reflect on healthy lifestyles, teach them strategies that facilitate risk communication, strengthen their self-confidence so that they can resist peer pressure and marketing appeals, and develop leadership skills to support peers who choose healthy lifestyles.

The multiplier students were trained using the following steps:

- The multiplier students were asked what they knew about the risks associated with inadequate physical activity, unhealthy diet, smoking, and alcohol consumption.
- Using the answers, the teachers compiled educational material that led smaller multiplier student groups to work through each of the themes.
- After working with the educational material, small groups of multiplier students planned interactions and activities to transmit the concepts to their peers, acquiring new and correcting erroneous concepts.
- The teachers encouraged the students to develop teaching methods (pedagogical dynamics) for working with the peer groups.
- The multiplier students constructed the pedagogical dynamics and presented them to the teachers, who then evaluated the content. Teachers corrected or commented on content only, preserving the proposed teaching methods.
- The teachers scheduled the activities of the multiplier students with their peers.
- The multiplier students led the activities with their peers.

The groups were located in 4 similar public schools. The cultural differences among the adolescents in each group were worked out without treating a particular culture as dominant or normative. For instance, the multiplier students compiled their mothers' recipes into a cookbook to make the point that a healthy diet can be created from the foods of any cultural tradition. The book included recipes from African, American, Arabian, Italian, Japanese, and other cultures.

Play (games and role-play) is essential to adolescent learning (8), and the multiplier students were allowed to use any type of language they wished to communicate with their peers. The student activities centered on the 4 themes of physical activity, healthy diet, smoking, and alcohol consumption.

## Sports Activities and Games: Physical Activity Theme

**Figure 1:**
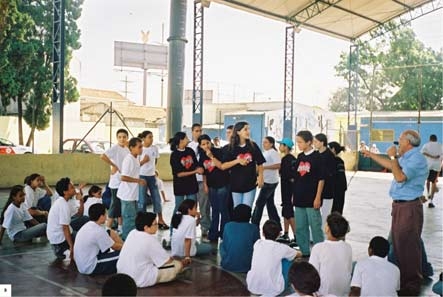
Multiplier students (in black shirts) organize a physical activity demonstration for younger peers.

During the training, the physical education teacher showed the multiplier group how to measure heart rate; this activity taught the group that the heartbeats of physically active people change more slowly than do those of inactive people. The group decided to demonstrate the heartbeat exercise by organizing a list of physical activities and inviting peers to participate (Figure 1).

The multiplier students used illustration boards to show the younger students how to perform the activities. Their goal was to demonstrate that physical activity could be fun and make people feel good; students participated in running and ball-tossing, a 15-minute soccer game, other ball games, hula hoop, jumping jacks, and skipping rope, among other aerobic activities, to establish the association between pleasure and physical activities. After these activities, multiplier students led a classroom discussion about the activities.

To control the content of the intervention, teachers observed the activities and evaluated them with the multiplier students. In evaluating the physical activity demonstration, the teachers noted the students' enthusiasm in associating enjoyable play with the benefits of physical activity.

## Food Demonstrations and Tasting: Healthy Diet Theme

**Figure 2:**
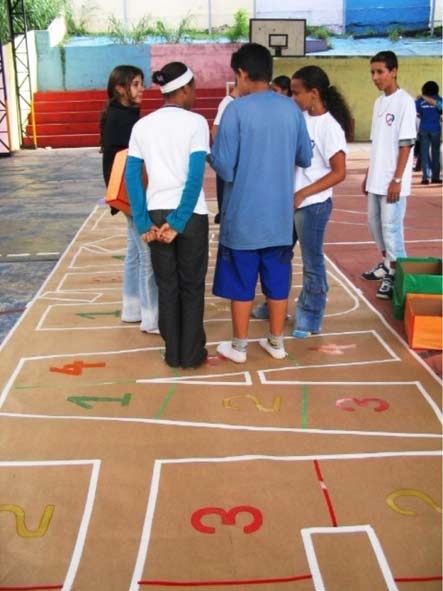
A hopscotch game was part of a lesson on healthy foods.

For the healthy diet theme, multiplier students chose a food demonstration in 4 phases. During the first phase, they talked about the benefits of a healthy diet. They displayed handmade posters throughout the school, calling attention to the colors of different foods and the importance of eating a variety of foods. In the second phase, they made posters with peers, using collage material from magazines, newspapers, and supermarket flyers, focusing on healthy and less healthy foods and daily activities. During this activity they talked with peers about lifestyles and habits. In the third phase, they provided foods for peers to taste as part of a hopscotch game with questions and answers (Figure 2). The game aimed not to test the peers' knowledge but to select foods, during a fun activity, for the peers to taste.

The peers made faces depending on the offered food, which entertained the students, and the participation achieved the intended goal of the content through simple and meaningful language. Teachers not engaged in the project reported that peers in the classroom talked about the taste of certain foods.

**Figure 3:**
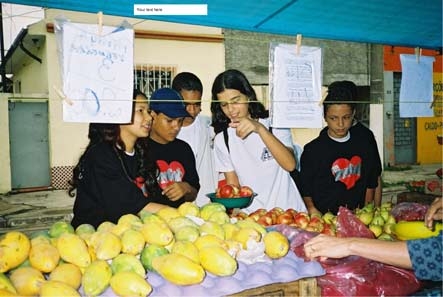
Multiplier students set up an open-air market to compare the prices of healthy foods with those of less healthy foods.

Another resource the students used was an open-air market (Figure 3), which allowed them to compare the prices of healthy foods with those of unhealthy foods such as fried snacks, cookies, and soft drinks.

## Theater and Music: Smoking Theme

Theater and music were important ways the multiplier students communicated information about smoking. Theater was the first form of communication the students used. This form allows the understanding that the meaningful and figurative aspects can only be taken together, a way of communication in which the healthy and unhealthy habits can talk with each other through music and dialogues between friends, cousins, and siblings. In the didactic activity, the following dialogue was used in the theatre:

Hey, Joe, dude, did you see John's uncle went to the hospital? He couldn't breathe any more, the aunt said he had a heart attack. Dude, he smoked so much it was impossible to breathe near him!

Yeah . . . and they say that when it is like that, we are passive smokers.

For real, man?

Yeah, there is a dude at school who said that cigarettes have more than 4000 toxic substances and that they spread in the smoke that enters the lungs and that burns at the tip of the cigarette, and if you are near it and breathe this smoke, we smoke too. And he's a great pal.

Really, dude, seriously?

This process aims to promote the cultural development and personal growth of the players through mastery and interactive use of theatrical language. The communication arises from the creativity and spontaneity of the interaction between individuals engaged in the dramatic solution of a health risk problem (9).

The theatrical activities comprised dialogues, poems, and songs. The songs, with lyrics adapted from familiar melodies, gave students the opportunity to use humor to present the concept of the healthy habit. The amount of interaction and level of student response indicated that live theater is an effective means of stimulating both thought and discussion pertaining to the effects of risk behavior (10).

During these activities, students sang "The Cigarette" to the melody of "Stupid Cupid" by Paul Anka, a song that became a hit in Brazil:

"Oh, Oh, cigarette, please leave me alone.

My heart can't take it anymore

I smoked till a while ago

Hey, hey, that's the end

Oh, cigarette, go far from me."

Vygotsky says that the process of concept formation is rooted in the use of words because they acquire different meanings in each of the phases of human mental development and activity. Thus, by singing with their peers, students move through different stages of learning about the themes (11).

## Puppet Theater: Alcohol Consumption Theme

**Figure 4:**
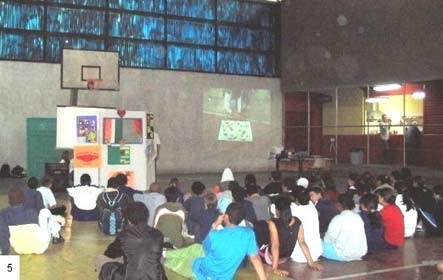
Students at a puppet theater performance on the topic of alcoholism.

Multiplier students chose a puppet theater (Figure 4) to teach peers about alcohol consumption. The students created a stage play with 5 performers, 4 of whom portrayed a family: a grandmother, 2 adolescents (a boy and a girl), and an uncle, who always appeared drunk, embarrassing the family and disrupting their daily life. The fifth character was a toucan that spoke with the audience, correctly answering questions about alcoholism. After the first performance, because of the large number of questions, such as whether physical activity helps to fight alcoholism, the student multipliers broadened the content of the play to include all 4 themes of the project.

Alcohol consumption was the most difficult topic for the students, many of whom have experienced, in their family or community, embarrassing situations caused by alcohol consumption. The use of the puppet theater allowed the students to develop a dialogue with the peers without embarrassment and, based on many individual reports, the audience participation attracted the attention of the entire school. The content of the puppet theater went beyond cardiovascular risks, including car accidents and fights caused by alcohol consumption.

## Board Games: Multiple Themes

The multiplier students developed games for each of the 4 themes to encourage discussion among the peers. Four players competed by throwing dice to determine the number of spaces to advance on a board. Each space had a number corresponding to a card, on which was written a question about the theme. The questions covered all 4 themes (physical activity, diet, smoking, and alcohol consumption). Players might be asked the following questions: Why is physical activity good for the heart? What do fats do to the heart? What does it mean to have foods of 5 different colors at one meal? Why does the health department warn against cigarette smoking? Do you know what the healthy habits for the heart are?

**Figure 5:**
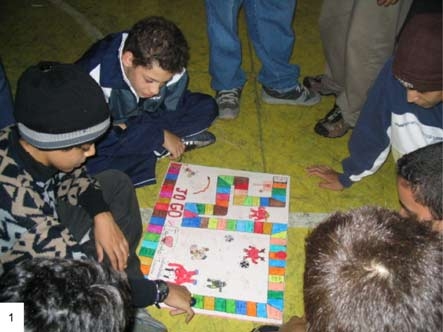
Peers play a board game.

Players who answered correctly advanced; players who answered incorrectly were penalized by backtracking 1 space. When a peer gave the wrong answer, the multiplier student asked whether anyone knew the right answer, and the one who did received a small reward. The multiplier students had decided on rewards such as apples and bookmarks (made by the students) with phrases that encouraged healthy habits. Key chains were offered to those who were penalized, so they would not forget the importance of treating the heart well (Figure 5).

Multiplier students succeeded because they did not allow the teaching to interfere with the design of the game. For example, the game penalized players for incorrect answers as part of the fun of the game. Even when teachers admit that playing is learning, they tend to set limits on the untamable, spontaneous expression of the students (12) and to give priority to the lesson.

The multiplier students felt comfortable explaining each answer and even used some technical terms, showing their familiarity with the subject. Furthermore, they were not embarrassed to say, "I don't know the answer." When players asked the multiplier students a question designed to confuse them, they requested assistance from one of the teachers (during the activity) or the research team (during the training).

During the playing of board games, we noticed that the students used our terminology in the questions. The games were as successful as the theatrical, musical, and food experimentation activities (Figure 2), but because they emphasized rote learning, the board games drew the attention of researchers to the concepts being transmitted rather than to the form of transmission, which did not occur with the forms of theater and music. The students were more creative in designing the game than they were in wording the questions.

We succeeded in our intention to allow the multiplier students to determine how to transmit the concepts. We tried to avoid a "don't do it" message because many adolescents and their family members smoke or drink alcohol, and such a message would push them further from the dialogue. Furthermore, we did not want to repeat the messages of other people, the media, and laws. We avoided the concepts of "right" and "wrong," and instead used the word "healthier." The techniques used should encourage students to reflect on their own choices regarding physical activity, diet, smoking, and alcohol consumption. The students did not repeat the conventional warnings such as "don't do it,"  "don't eat it," "stop doing it for your own good," and "it is bad for your health," which was one of our goals. It was satisfying to the technical team and the teachers, who, during the training sessions of the multiplier students, many times had to counter media portrayals and common beliefs about health risk behaviors (for example, those that glamorize smoking). When designing the educational activities used in this project, we looked mainly at activities that would empower students and their peers (13).

Multiplier students at one school requested space for a Multiplier Students Corner at the science fair, so the whole school could be made aware of the program. They set up posters, which they and their peers had made, describing the program themes and the history of the program. This space promoted reflection on the themes by the school community and school visitors.

The Healthy Lifestyle Multiplier Students program prompted the community to discuss themes that were absent from the school's formal curriculum. The proposed health education process was creative and motivational and encouraged the adolescents to learn about health while improving the quality of public education.

We recommend that those who are interested in creating such programs gather the students, show them the content, allow them to determine the process, and follow the development of the process among peers. We encourage others to foster adolescents' capacity for leading the teaching-learning process.

## Supplementary Material

Supporting Video 1 Watch a short video about the Healthy Lifestyle Multiplier Students Program **Part 1** (WMV, 7.1Mb)

Supporting Video 2 **Part 2** (WMV, 4.26Mb)

Supporting Video 3 **Part 3** (WMV, 4.13Mb)

Supporting Video 4 **Part 4 **(WMV, 4.85Mb)

Supporting Video 5 **Part 5** (WMV, 3.12Mb)

## Appendix.

### Course Syllabus for Improving Teachers' Skills

1. The role of the multiplier students
2. Healthy lifestyle and CVD
3. The practice of physical activity and its benefits
4. Healthy diet
5. Hazards of smoking
6. Hazards of alcohol consumption
7. Cultural, social, psychological, and anthropological features of habits acquisition
8. Diversity — distinct diets in Brazil
9. CVDs
  - Prevalence and risk
  - Arterial hypertension
  - Good and bad cholesterol
  - Hazards of diabetes
  - Genetic factors
10. The pedagogical methods and practices
  - Paulo Freire's dialogical praxis
  - Lev Vygotsky's concept of development
  - Jaime Moreno's games and learning
  - Learning how to learn, learning how to think
  - Beginning with the previous knowledge
  - Allowing adolescent creativity
  - Learning autonomy
  - Working with group language
